# Dye Decolourisation Using Two *Klebsiella* Strains

**DOI:** 10.1007/s11270-014-2249-6

**Published:** 2014-12-11

**Authors:** Ewa Zabłocka-Godlewska, Wioletta Przystaś, Elżbieta Grabińska-Sota

**Affiliations:** Environmental Biotechnology Department, Silesian University of Technology, Akademicka 2A, 44-100 Gliwice, Poland

**Keywords:** *Klebsiella*, Azo dyes, Triphenylmethane dyes, Dyes mixture, Decolourisation, Zootoxicity, Phytotoxicity

## Abstract

This study aimed to decolourise different dyes using two *Klebsiella* strains (Bz4 and Rz7) in different concentrations and incubation conditions. Azo (Evans blue (EB)) and triphenylmethane (brilliant green (BG)) dyes were used individually and in mixture. The toxicity of the biotransformation products was estimated. Both strains had a significant potential to decolourise the dyes in the fluorone, azo and triphenylmethane classes. The type and concentration of dye affects the decolourisation effectiveness. Differences in the dye removal potential were observed particularly in the main experiment. The best results were obtained for Bz4 in the samples with EB (up to 95.4 %) and dye mixture (up to 99 %) and for Rz7 with BG (100 %). The living and dead biomass of the strain Bz4 highly absorbs the dyes. Significant effect of the process conditions was noticed for both strains. The best results were obtained in static and semistatic samples (89–99 %) for the removal of EB and a mixture of dyes and in static samples (100 %) for BG. The decrease in zootoxicity (from class IV/V) was noticed in all samples with living biomass of the strain Bz4 (to class III/IV) and in samples with single dyes for Rz7 (to class III/IV). The decrease in phytotoxicity (from class III/IV) was noticed for Bz4 in the samples with BG and a mixture (to class III) and for Rz7 in the samples with BG (to class III). The process conditions did not affect the changes in toxicity after the process.

## Introduction

Azo and triphenylmethane (TPM) dyes are classes of synthetic dyes that are used in many industrial branches for dyeing, e.g. wool, cotton, nylon, leather, paper, cosmetics, pharmaceuticals, food, plastic and petroleum products (An et al. 2002; Cui et al. 2012; Franciscon et al. 2009a, b; Hamid and Rehman 2009; Koyani et al. 2013; Padamavathy et al. 2003; Somasiri et al. 2006; Swamy and Ramsay 1999; Tony et al. 2009; Younes et al. 2012).

The common usage of dyes causes an increase of their concentration in the environment. The disposal of wastewater with high dye concentrations leads to contamination of surface waters, reduces light penetration and photosynthetic activity and causes oxygen deficiency.

A serious ecological problem is connected with the persistence of dyes, their toxicity, mutagenicity and resistance to biological degradation because of their synthetic origin and complicated molecular structures. They are gradually accumulated in the food chain (Azmi et al. 1998; Banat et al. 1996; Forgacs et al. 2004; Franciscon et al. 2009a, b; Hamid and Rehman 2009; Koyani et al. 2013; Pointing and Vrijmoed 2000; Sani and Banerjee 1999; Wong and Yuen 1998). This situation requires the development of an environmentally friendly and economically acceptable treatment technology (Eichlerova et al. 2006; Hamid and Rehman 2009; Hu 2001; Koyani et al. 2013; Tony et al. 2009). Biological treatment based on microbial activity is more attractive than physicochemical methods. Biodegradation is considered an environmentally friendly and cost-effective option (An et al. 2002; Chen et al. 2003; Cui et al. 2012; Franciscon et al. 2009a, b; Wang et al. 2012; Wu et al. 2009; Wu et al. 2012).

Various organisms can remove and even mineralise dyes (bacteria, fungi, algae and plants) (Cui et al. 2012; Franciscon et al. 2009a, b; Hamid and Rehman 2009; Koyani et al. 2013, Srinivasan and Viraraghavan 2010; Tony et al. 2009; Younes et al. 2012). The most important factors that affect the efficiency of biological dye removal are the type of organisms, process conditions (e.g. temperature, oxygen level, pH and additional available carbon and nitrogen sources) and concentrations and the chemical structures of the dyes (Azmi et al. 1998; Banat et al. 1996; Cui et al. 2012; Franciscon et al. 2009a, b; Forgacs et al. 2004; Hamid and Rehman 2009; Koyani et al. 2013; Padamavathy et al. 2003; Pointing and Vrijmoed 2000; Przystaś et al. 2009; Sani and Banerjee 1999; Saratale et al. 2011; Stolz 2001; Swamy and Ramsay 1999; Tony et al. 2009; Younes et al. 2012; Zabłocka-Godlewska et al. 2009). Dye removal based on microbial activity is widely described. These technologies are based on the biodegradation/biotransformation of dyes using living bacteria and fungi and the physical sorption on living or dead biomass (An et al. 2002; Chen et al. 2003; Cui et al. 2012; Franciscon et al. 2009a, b; Wang et al. 2012; Wu et al. 2009; Wu et al. 2012).

Single strains of different bacterium taxonomic groups and their co-cultures and mixtures are used in dye removal technologies. The most popular genera of bacteria described in the literature are *Pseudomonas*, *Bacillus*, *Sphingomonas*, *Aeromonas*, *Citrobacter*, *Escherichia*, *Desulphovibrio*, *Proteus*, *Schewanella*, *Klebsiella*, *Alcaligenes*, and *Streptococcus* (An et al. 2002; Cui et al. 2012; Franciscon et al. 2009a, b; Nigam et al. 1996; Saratale et al. 2011; Sharma et al. 2004; Tony et al. 2009; Wang et al. 2012). *Klebsiella*, which are rod-shaped, non-motile, Gram-negative bacteria, have a thin cell wall that contains approximately 10 % peptidoglycan and outer membrane (contains proteins, phospholipids and lipopolysaccharides). Bacteria of this genus have a polysaccharide capsule and are oxidase negative (Salyers and Whitt 2000; Schlegel 1993). The decolourisation results of azo dyes in aerobic conditions by the *Klebsiella pneumoniae* strain were described by Wong and Yuen (1998). Cui et al. (2012) reported that a consortium of 13 bacterial strains including 11 isolates of *Klebsiella* sp. could significantly decolourise azo dyes in aerobic and anaerobic conditions. The decolourisation of azo dyes by *Klebsiella pneumonia*e was also studied by Zhang et al. (2010), Franciscon et al. (2009b) and Elizalde-Gonzales et al. (2009).

Most existing studies on dye removal concentrate on individual dyes (mostly the azo class) although they are present in wastewater as a mixture. There remains a lack of information about the efficacy of decolourisation of dye mixtures that contain dyes from different groups. In our study, we focused on the removal efficiency of various dyes (azo Evans blue and triphenylmethane brilliant green) individually and in mixture using two different strains from the *Klebsiella* genera. A notably important part of the decolourisation studies is the assessment of environmental risk, which is connected with the possibility of producing toxic biotransformation products. Our study evaluated the toxicity of biotransformation products on aquatic organisms.

## Materials and Methods

### Isolation of Bacterial Strains

The bacteria that were used in the experiment were isolated from the inlet of the wastewater treatment plant in Katowice (Bz4) and the polluted river Leśnica in Wodzisław Śląski (Rz7) located in south Poland. The wastewater treatment plant in Katowice has a daily flow of approximately 43,000 m^3^ day^−1^. According to Polish law (Dz. U. 2012 r. poz.145; Dz. U. Nr257, poz. 1545), Leśnica river is classified as a river with poor ecological status.

The bacteria strains were isolated using the spread plate method on nutrient agar (BTL), which was supplemented with 0.1 g l^−1^ of brilliant green. After 72 h of incubation, a discolouration zone was observed around some bacterial colonies, which proved their decolourisation abilities. The colonies with the largest discoloured area, which was measured from the edge of the colony (11 and 18 mm for Bz4 and Rz7, respectively), were isolated and purified on nutrient agar (BTL) using the streak plate method. Selected strains were classified as *Klebsiella planticola* (Rz7) and *Klebsiella* sp. (Bz4) using the API 20E test (Biomerieux).

### Decolourisation Potential

In the experiment, the Kimura medium (pH 6.8) was used (Zabłocka-Godlewska et al. 2012). Bacterial suspensions for medium inoculation were prepared in physiological salt (the optical density of each suspension was ∼15 × 10^8^ cfu ml^−1^). Then, 0.1 ml of innoculum was added to tubes that contained 10 ml of the medium. Five different dyes (fluorone: Bengal rose (BR), erythrosine (E); triphenylmethane: brilliant green (BG), crystal violet (CV); azo: Evans blue (EB)) in two concentrations (0.05 and 0.1 g l^−1^) were used.

Filter-sterilised dyes were added to tubes with 48-h-old bacteria cultures (stationary growth phase). All samples were prepared in triplicate. The samples were incubated at 26 °C for 144 h. After the experiment ended, the samples were collected and centrifuged (5000 rpm/10 min), and the supernatant was used for absorbance estimation on a spectrophotometer (UV–Vis Hitachi U-1900). The maximal absorbance was experimentally determined for each dye (527 nm for erythrosine, 548 nm for Bengal rose, 590 nm for crystal violet, 624 nm for brilliant green and 606 nm for Evans blue) (Zabłocka-Godlewska et al. 2012). The percentage of dye removal was calculated according to formula 1.

1 $$ \mathrm{R}=\left(\mathrm{C}\hbox{-} \mathrm{S}/\mathrm{C}\right)\times 100\% $$

R—Dye removal (%)

C—Dye concentration in a control sample (mg l^−1^)

S—Dye residue concentration in a sample with living/dead bacteria biomass (mg l^−1^)

### Main Experiment

The dye concentration in the main experiment was determined based on the concentration test. The effect of five different concentrations (0.01, 0.025, 0.05, 0.075 and 0.1 g l^−1^) of Evans blue and brilliant green on the decolourisation effectiveness was estimated. For the dye mixture, an equal proportion (1:1) of brilliant green and Evans blue was used. The effect of six different mixture concentrations (0.02, 0.04, 0.06, 0.08, 0.1 and 0.12 g l^−1^) on the decolourisation effectiveness was studied. An identical experiment was performed for the liquid Kimura medium (pH 6.8).

Inoculum and dyes were prepared using the method described in Sect. 2.2, except that the inoculum was added to 100-ml flasks that contained 50 ml of Kimura medium. The samples were incubated for 6 days at 26 °C. The samples were collected and centrifuged (5000 rpm/10 min), and the supernatant was used to estimate the absorbance. The absorbance was measured on a UV–Vis Hitachi U-1900 at determined wavelengths for Evans blue (λ max, 606 nm), brilliant green (λ max, 624 nm) and the dye mixture (λ max,591 nm). The percentage of dye removal was calculated according to formula 1 (Sect. 2.2).

The main experiment was performed using the same method as in our previous study on *Pseudomonas* strains (Zabłocka-Godlewska et al. 2014). For this part of the experiment, the dye concentrations were selected based on the results of the concentration test (0.05 g l^−1^ for Evans blue; 0.1 g l^−1^ for brilliant green; 0.08 g l^−1^ for the mixture). For the mixture, Evans blue and brilliant green were prepared in a ratio of 1:1 (0.04 g l^−1^ of BG and 0.04 g l^−1^ of EB). Identically to the previous case (Zabłocka-Godlewska et al. 2014), the cultures were incubated in different conditions (static, semistatic and shaken). The shaken samples were shaken on a rotary shaker for 24 h day^−1^ (200 rpm); the semistatic samples were shaken with the same speed as the shaken samples every other day. The static samples were not shaken at all. To determine the degree of sorption, autoclaved dead biomass was used.

Samples were taken after 1, 6, 24, 48, 72, 96 and 120 h of incubation and centrifuged (5000 rpm/10 min), and the supernatant was used to estimate the absorbance using a UV–Vis spectrophotometer Hitachi U-1900. The percentage of dye removal was calculated as previously according to formula 1.

### Toxicity Evaluation

*Daphnia magna* (OECD 202) was used to evaluate the zootoxicity. The phytotoxicity test was performed according to the OECD *Lemna* sp. growth inhibition test no. 221. All tests were performed in quadruple. After the estimation of EC_50_, the acute toxicity unit (TUa) was calculated (Zabłocka-Godlewska et al. 2014), which allowed us to establish a toxicity class. According to ACE 89/BE 2/D3 Final Report Commission EC, TUa < 0.4 corresponds to class I (non-toxic), 0.4 ≤ TUa < 1.0 corresponds to class II (low toxicity), 1.0 ≤ TUa < 10 corresponds to class III (toxic), 10 ≤ TUa ≤ 100 corresponds to class IV (high toxicity), and TUa > 100 corresponds to class V (extremely toxic).

## Results and Discussion

### Decolourisation Potential

This study concentrates on the effectiveness of dye decolourisation using two *Klebsiella* strains isolated from two different sites. Bacteria from the genus *Klebsiella*, which is part of the family “*Enterobacteriaceae*”, are opportunist pathogens that are responsible for human nosocomial infections. These infections are mainly caused by two strains: *Klebsiella pneumoniae* and *Klebsiella oxytoca* (Jonas et al. 2004; Podschun et al. 2000; Westbrook et al. 2000). In this experiment, we used *Klebsiella planticola* and *Klebsiella* sp. Isolated from the river, *K. planticola* (percentage of identification by API 20 E id% = 99 %) is restricted to aquatic, soil and botanic environments and rarely reported as the cause of human infections. The identification of *Klebsiella* sp. (isolated from the inlet of a wastewater plant) was more complicated. The numerical code and additional tests identify two strains: *K. planticola* and *K. terrigena*. Both strains are more related to each other than to *K. pneumoniae*. The strain *K. terrigena*, like *K. planticola*, is an environmental strain that is mainly isolated from soil and water but rarely from humans or clinical specimens. Literature data shown low virulence of the aforementioned *Klebsiella* strains (Boye and Hansen 2003; Jonas et al. 2004; O’ Connell et al. 2010; Olson et al. 2013; Podschun et al. 2000; Westbrook et al. 2000; Zlotnikov et al. 2001). Their usage in bioremediation processes appears relatively safe for the environment and public health.

The results of the decolourisation potential test (Table 1) show the differences between the abilities of both tested *Klebsiella* strains. Fluorone dyes (erythrosine, Bengal rose) and azo dye (Evans blue) were removed by both strains at comparable levels (from 86 % at high concentration to 100 % at low concentration). Differences were observed in the removal effectiveness of triphenylmethane dyes (brilliant green and crystal violet) at higher concentration (0.1 g l^−1^). Bz4 removed up to 81.4 % of BG and 59.8 % of CV, whereas the strain Rz7 removed more than 98 % of both dyes. As observed, the decolourisation potential is related to the strain, dye concentration and structure, which was also noticed in other studies. Our obtained results for two *Pseudomanas* strains (Zabłocka-Godlewska et al. 2012) proved that the dye structure and concentration and the site of the strain isolation affect the decolourisation effectiveness. The *Pseudomonas fluorescens* strain Sz6 almost completely removed TPM dyes (crystal violet and brilliant green) regardless of the dye concentration, whereas the *P. fluorescens* strain SDz3 removed approximately 90 % of these dyes only at the concentration of 0.05 g l^−1^. The removal results of azo Evans blue for *Klebsiella* strains (Table 1) were comparable with the results of Zabłocka-Godlewska et al. (2012) for the *Pseudomonas* strain Sz6, but they were much lower for the strain SDz3 (<73 %). Fluorone dyes were more than 85 % removed (Table 1) by both tested *Klebsiella* strains, whereas in our previous study on *P. fluorescens* (Zabłocka-Godlewska et al. 2012), the strain SDz3 removed them at comparable levels and the strain Sz6 removed less than 55 %. Alhassani et al. (2007) proved that *Brevibacillus* sp. isolated from Coomassie Brilliant Blue (triphenylmethane dye) polluted soil had high decolourisation potential. Among eight tested dyes from different groups, only three were not removed by this strain. Wu et al. (2009) tested the decolourisation potential of TPM and azo dyes by *Pseudomonas otitidis* WL13 when different dye concentrations were present in the samples. The tested strain showed the highest decolourisation capability against TPM malachite green and brilliant green. Both dyes were removed by more than 95 %, even at a concentration of 500 μmol l^−1^. Crystal violet was completely removed at concentrations of up to 20 μmol l^−1^, 65.5 % removed at a concentration of 200 μmol l^−1^ and only 13.2 % removed at a concentration of 500 μmol l^−1^. TPM were better removed than azo dyes, which is related to the more complex structure of dyes from the azo class. In the study of Wu et al. (2009), the dyes with the monoazo group were better removed than diazo. Cui et al. (2012) tested the effectiveness of azo dye removal using a consortium composed of different *Klebsiella* strains. Eriochrome black T and methyl orange were less than 80 % removed after 16 h, but the other four azo dyes were completely removed. Zhang et al. (2010) tested eight different bacteria consortia for the decolourisation of 14 azo dyes. Unlike the hydroxyl group, the presence of carboxyl and sulfo groups significantly increased the azo dye biodegradability.

**Table 1:** Results of the decolourisation potential test

Dye removal (%)	Strain Bz4		Strain Rz7	
	Initial dye concentration			
	0.05 g l^−1^	0.1 g l^−1^	0.05 g l^−1^	0.1 g l^−1^
Erythrosine	99.02 ± 1.62	86.0 ± 0.5	100.66 ± 0.81	93.9 ± 1.0
Bengal rose	100.08 ± 1.96	100.00 ± 0.0	100.02 ± 1.11	93.3 ± 0.92
Crystal violet	98.8 ± 1.73	59.8 ± 0.44	99.00 ± 0.49	98.6 ± 0.43
Brilliant green	100.55 ± 0.62	81.4 ± 0.4	100.07 ± 0.13	98.0 ± 0.2
Evans blue	98. 79 ± 1.81	86.01 ± 0.81	99.52 ± 0.34	90.5 ± 0.4

### Main Experiment—Decolourisation of Dyes

#### Effect of the Dye Concentration on the Effectiveness of Dye Decolourisation

**Fig. 1:**
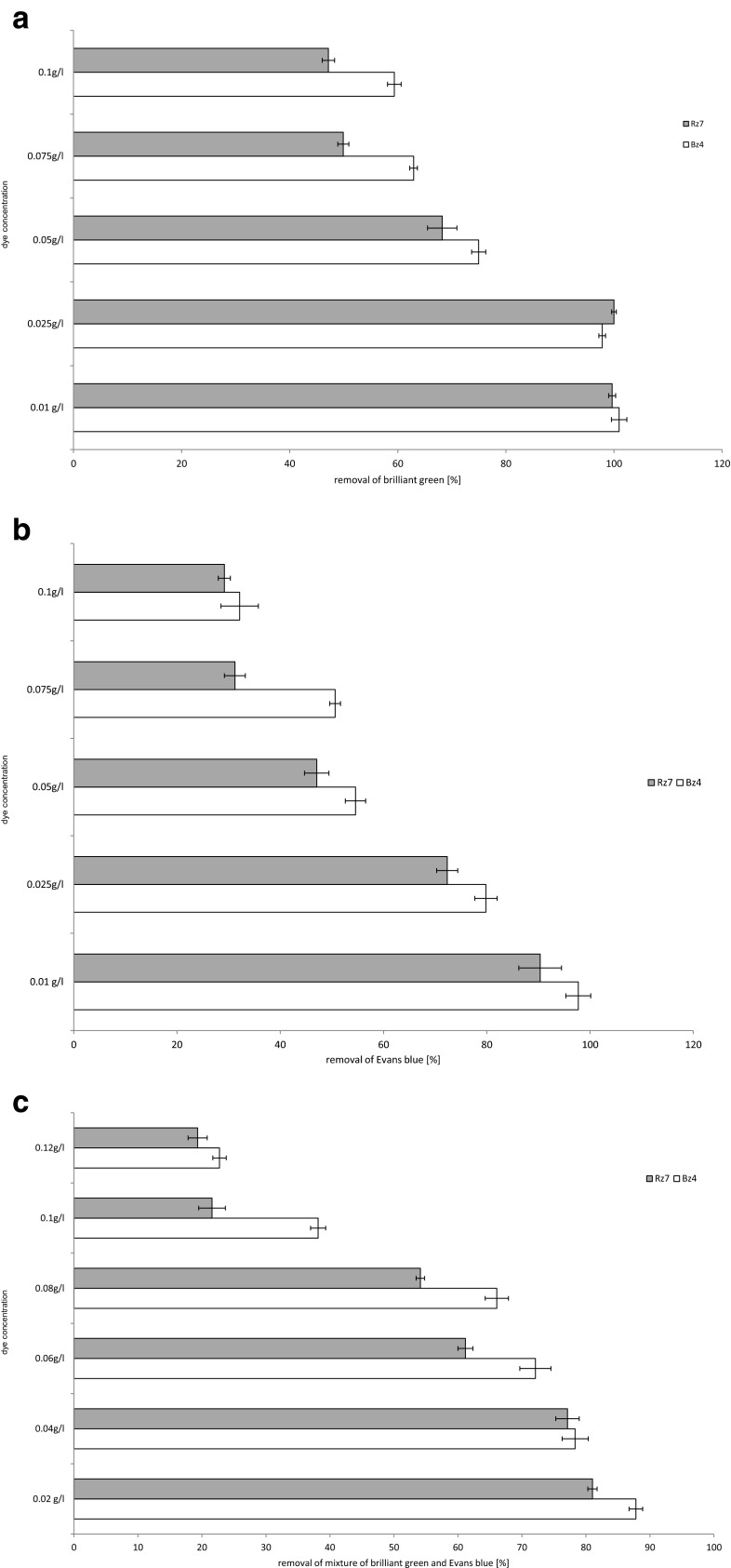
Influence of brilliant green (**a**), Evans blue (**b**) and the dye mixture (**c**) concentration on the effectiveness of their removal

The dye concentrations for the main experiment were selected based on the concentration test (Fig. 1a–c). When the brilliant green concentration increased to 0.05 g l^−1^ and more, the decolourisation effectiveness of both tested strains (Fig. 1a) decreased. After 6 days, the removal of BG in a sample with a concentration of 0.05 g l^−1^ was 74.96 % for the strain Bz4 and 68.22 % for Rz7. The increase in concentration to 0.075 g l^−1^ resulted in further decrease of dye decolourisation effectiveness (62.92 and 49.91 %, respectively). At the highest concentration of BG (0.1 g l^−1^), the obtained dye removal was 59.36 % for Bz4 and 47.16 % for Rz7. Higher dye concentrations in the sample are more toxic for bacteria. The strain tolerance to xenobiotics is related to their individual properties (e.g. the sorption potential which is connected with the cell wall structure, presence of a capsule or specific enzymatic system). The obtained results in our previous study (Zabłocka-Godlewska et al. 2014) for two *Pseudomonas* strains showed better tolerance to higher BG concentrations than *Klebsiella* strains (Fig. 1a). The effect of the initial dye concentration on the dye removal effectiveness was documented by Wu et al. (2012). For *Shewanella oneidensis* MR-1, the decolourisation ability gradually decreases with the increase in dye concentration. The removal of brilliant green and malachite green (TPM) by *Citrobacter* sp. was studied by An et al. (2002). After 1 h, both triphenylmethane dyes were completely removed at concentrations of 5 and 10 μM. An increase in concentration to 100 μM decreased the decolourisation to ∼82 % and further increase in dye concentration resulted in further decrease of the removal level.

Evans blue had worse decolourisation test results than brilliant green (Fig. 1b). A similar effect was found for the previously studied *Pseudomonas* strains, particularly in the case of SDz3 (Zabłocka-Godlewska et al. 2012, 2014). Diazo Evans blue appears to be a more difficult substrate for the studied strains. After 6 days of experiment in the sample with the lowest concentration (0.01 g l^−1^), the dye removal reached 90.72 % for Bz4 and 90.34 % for Rz7 strain. In addition, in this case, at higher dye concentration, the decolourisation effectiveness decreased. In samples with 0.05 g l^−1^ of EB, the removal was 54.56 % for Bz4 and 47.07 % for Rz7. At the highest concentration (0.1 g l^−1^), the dye removal was only 32.14 and 29.17 % for Bz4 and Rz7, respectively. An et al. (2002) studied the decolourisation of diazo dye Congo Red using *Citrobacter* sp. An increase in concentration to 50 and 100 μM resulted in a strong decrease in decolourisation efficacy to 28 and 26 %, respectively. At the concentration of 2000 μM, no decolurisation of Congo Red was observed. Isik and Sponza (2003) conducted an experiment with *Escherichia coli* and reached 100 and 85 % removal of Congo Red when the dye concentration varied between 250 and 2500 mg l^−1^, whereas 55 % removal was obtained for 3000 mg l^−1^. Cui et al. (2012) presented the results of methyl red removal using a consortium of bacteria (mostly consisted of *Klebsiella* strains). In anaerobic conditions, the decolourisation was almost complete (94–98 %) after 10 and 20 h when the dye concentration was 200 and 500 mg l^−1^, respectively. In aerobic conditions, the consortium required 40 h to remove 95 % of 200 mg l^−1^ MR and 48 h to remove 70 % at a concentration of 500 mg l^−1^.

A potential decolourisation test was performed for a mixture of both dyes, which shows that at the lowest tested concentration, the removal was less than 90 % when the single dyes were almost completely removed (Fig. 1c). At a concentration of 0.08 g l^−1^, the dye removal was 66.08 % by Bz4 and 54.13 % by Rz7. The decolourisation effectiveness of both strains significantly decreased when the dye concentration was 0.1 g l^−1^ (38.16 and 21.57 %, respectively). The effect of the dye mixture concentration on the decolourisation effectiveness is not well documented in the literature. A similar tendency was found for *Pseudomonas* strains that were previously studied (Zabłocka-Godlewska et al. 2014). Apart from our previous work, there is a lack of information about the effect of the concentration when the mixture of dyes is composed of dyes of different classes. Vijaykumar et al. (2007) studied the decolourisation efficacy of *Kerstersia* sp. for mixtures of three azo dyes (Amaranth, Fast red E, Ponceau S) at five concentrations. The concentrations of 200–800 mg l^−1^ were completely removed, but the required time for their removal was longer when the dye content increased. Based on the results of our test, different concentrations were used for further studies: 0.1 g l^−1^ of brilliant green, 0.05 g l^−1^ of Evans blue and 0.08 g l^−1^ of mixture (0.04 g l^−1^ of BG and 0.04 g l^−1^ of EB).

#### Effect of the Growth Conditions on the Delcolourisation Effectiveness—Main Experiment

The main experiment was performed under static, semistatic and shaken conditions. The sorption was estimated using bacteria dead biomass. The usage of dead biomass allows an approximate determination of the biomass sorption properties with the exclusion of biotransformation and/or biodegradation. The mechanism of microbial decolourisation occurs from adsorption, enzymatic degradation or a combination of both. The obtained results using the samples with dead and living biomass must be compared to specify the participation of both mechanisms in dye decolourisation.

**Fig. 2:**
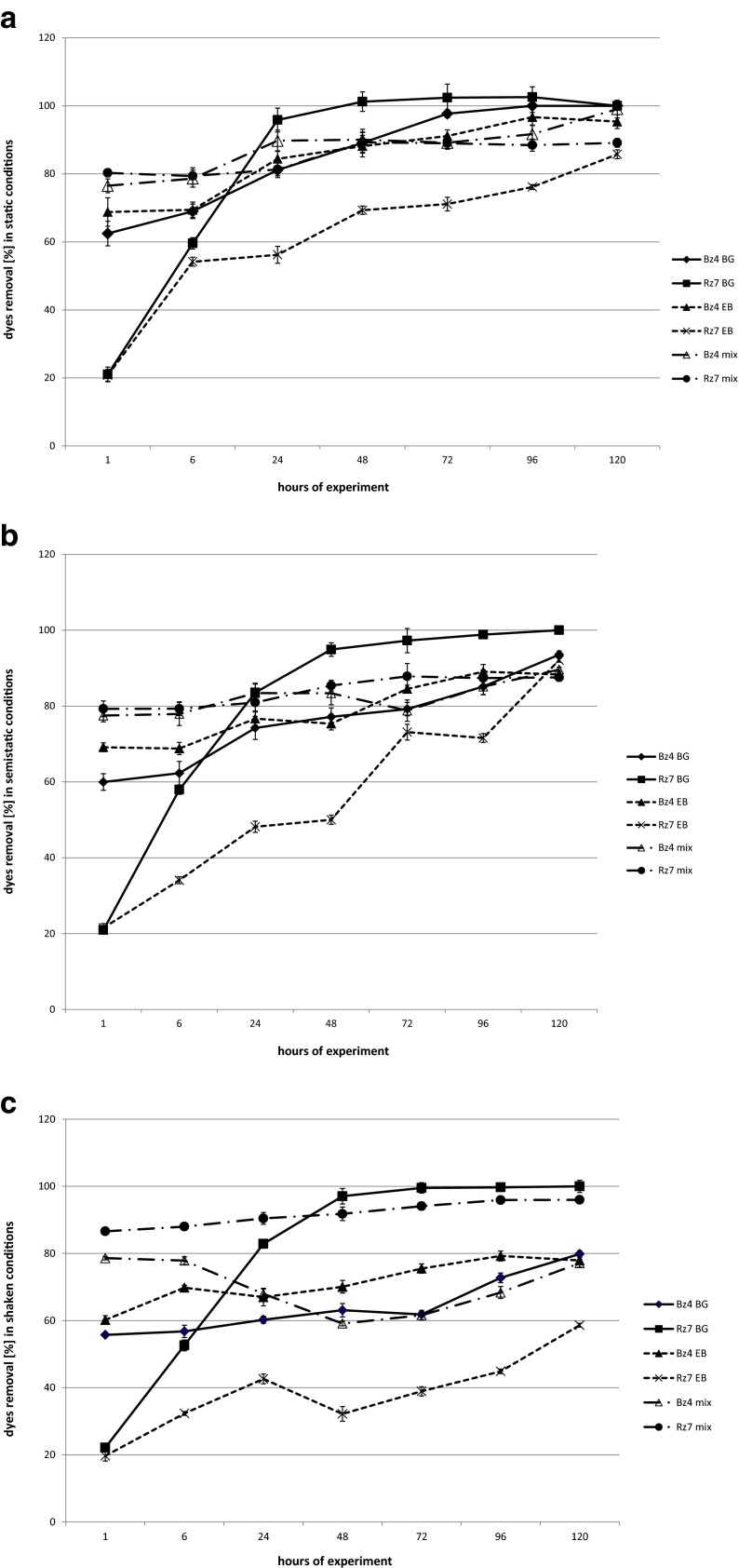
Percentage of removal of the dyes in the static (**a**), semistatic (**b**) and shaken samples (**c**)

**Fig. 3:**
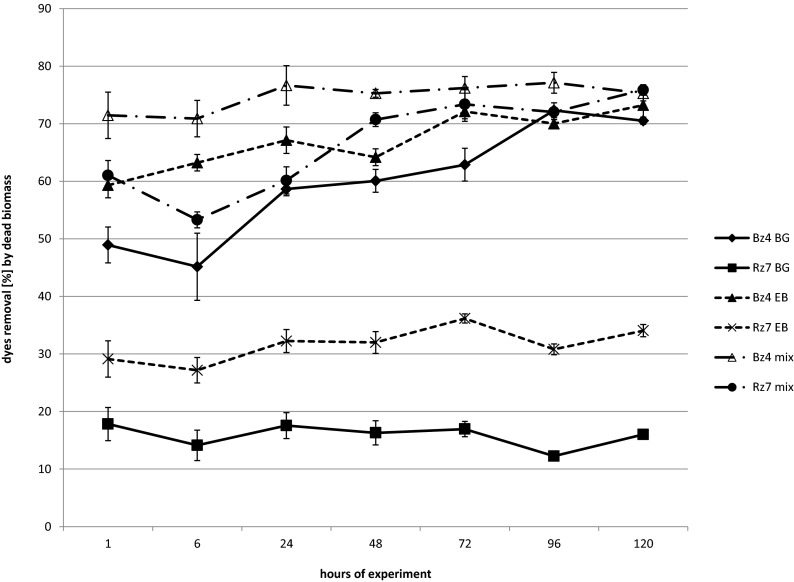
Removal of the dyes in samples with dead biomass

Brilliant green was better removed by the strain Rz7 than Bz4 regardless of the culture conditions (Fig. 2a–c). In the sample with Rz7, almost complete decolourisation was obtained under static conditions after 24 h. Identical levels of dye removal in semistatic and shaken conditions were obtained after 48 h of experiment. The brilliant green removal by the strain Bz4 in static conditions after 24 h was 81.14 %, and all dye was removed after 96 h of experiment. Worse results for this strain were noticed in the semistatic condition and particularly in the shaken condition. Shaking increases the dissolved oxygen concentration, evenly distributes it throughout the sample and transfers it between the cells and the medium. In the shaken samples, the bacteria have better contact with the dyes, which should positively affect decolourisation. The previously presented results note that shaking negatively affects the dye removal rate, particularly during first hours of the experiment. These results confirm that facultative anaerobic strains of *Klebsiella* prefer lower oxygen concentration. Finally (at 120 h), all BG was removed by the strain Bz4 in the static samples, 93.56 % in the semistatic samples and 79.85 % in the shaken samples. It should be noted that despite the end results, the strain Bz4 has high effectiveness of BG removal (approximately 60 %) after the first hour of the experiment regardless of the process conditions, which is related to the high sorption potential of this strain. The biosorption capacity of microorganisms depends on the composition of the cell wall (heteropolysaccharides and lipids). These components contain different functional groups (amino, carboxyl, hydroxyl, phosphate and other charged groups), which cause strong attractive forces between the dye molecule and the bacteria cell wall. The results obtained for the dead biomass (Fig. 3) and observed intensive tint of living biomass confirm this mechanism. After 1 h of the experiment, the dead biomass of Bz4 sorbed almost 50 % of BG (and 70 % after 120 h), whereas the strain Rz7 removed less than 20 %. These results suggest a higher contribution of biodegradation processes in BG decolourisation by the strain Rz7.

The pathways of bacterial biodegradation of triphenylmethane dyes remain not well documented. There is lack of information about the dye metabolic pathway for the genus *Klebsiella*. Jang et al. (2005) studied *Citrobacter* sp. strain KCTC18061P which belongs, the same as *Klebsiella*, to *Enterobacteriaceae* family. The reported strain had a high decolourisation potential of triphenylmethane dyes. The enzyme that is responsible for the decolourisation abilities is triphenylmethane reductase (TMR). Wang et al. (2012) proposed a pathway of methyl green (MG) degradation by *Exiguobacterium* sp. MG2 under shaking conditions. The mechanisms of this dye degradation were related to the activity of TMR and cytochrome P450. The first step was connected with the TMR activity, which catalyses MG into its leucoform, which is later transformed by cytochrom P450. The decolourisation efficiency was more than 90 % after 3 h of experiment for all tested concentrations (1000–2500 mg l^−1^). Wang et al. (2011) proved that during the malachite green decolourisation by *Achromobacter xolosoxidans* MG1, extracellular enzymes and some heat resistance extracellular compounds were involved. The incubation conditions (static and shaking) did not significantly affect the dye removal. The decolourisation of crystal violet (TPM dye) at a concentration of 30 μmol l^−1^ was studied by Wu et al. (2009). After 36 h, the decolourisation by *Pseudomonas otitidis* was approximately 20 % under static conditions and less than 15 % under shaking conditions. For this strain, the dye adsorption on biomass was observed. In other experiment presented by the same authors, crystal violet was almost completely removed in shaking conditions after 12 h at concentrations of up to 100 μmol l^−1^. An et al. (2002) studied the decolourisation of crystal violet at a concentration of 20 mg l^−1^ under various conditions. The best results were achieved in the shaken samples. After 4 h of incubation, almost complete removal was observed in the shaking samples, whereas it was less than 40 % in the static samples. After 16 h, complete removal was observed regardless of the process conditions.

Both *Klebsiella* strains decolourised Evans blue with higher efficacy in static samples (Fig. 2a) than in shaken samples (Fig. 2c). In static and shaken samples, the strain Bz4 had better final decolourisation results (95.36 and 77.93 % removal, respectively) than the strain Rz7 (85.76 and 58.62 %, respectively). The final removals in semistatic conditions for both strains were at similar levels (∼90 %) (Fig. 2b). Similar to the aforementioned brilliant green results, shaking negatively affects the Evans blue removal rate, particularly during the first hours of the experiment. Similar to the experiment with BG, the strain Bz4 removed 60–70 % of EB in the first hour of the test regardless of the culture conditions. As previously mentioned, these results and the results obtained in the samples with dead biomass confirm the high contribution of sorption in the EB removal by the strain Bz4, particularly at the beginning of the decolourisation process. The dead biomass of the strain Bz4 finally adsorbed up to 73.23 % of dye, whereas Rz7 adsorbed only 34.05 %. In our previous study with Evans blue and *Pseudomonas* strains, the effect of agitation on the decolourisation effectiveness was also negative. In static conditions, almost complete decolourisation was obtained by the strain Sz6 after 48 h, whereas in shaking conditions, it was less than 70 % (Zabłocka-Godlewska et al. 2012). In case of another *Pseudomonas* strain (SDz3), at the end of the experiment, the results were comparable.

The effects of oxygen and agitation on the azo dye decolourisation are widely described (Van der Zee and Villaverde 2005; Saratale et al. 2010; Saratale et al. 2011; Pearce et al. 2003). It is reported that during the bacterial decolourisation of azo dyes, both oxidative and reductive enzymes play a role (Pearce et al. 2003; Saratale et al. 2011). The first step of azo dye decolourisation is reduction by azoreductases, which are almost exclusively anaerobic in nature, so their activity is higher under anaerobic conditions, which is related to the competition for substrate between oxygen and azoreductases. However, a small amount of oxygen is required for the oxidative enzymes that are involved in the degradation of azo dyes. Franciscon et al. (2009a, b) studied the decolourisation of four azo dyes by *Staphylococcus arlettae* and *Klebsiella* sp. under microaerophilic and aerobic conditions. There was no significant difference in the final dye removal regardless of the conditions. Complete removal or almost complete removal (∼95 %) of all tested dyes was obtained for the tested *Klebsiella* strain after 168 h regardless of the incubation conditions. Elizalde-Gonzales et al. (2009) studied the degradation of immobilised azo dyes by *Klebsiella* sp. UAP-b5. After 48 h, effective decolourisation of the dye BB41 was observed in anaerobic conditions. Cui et al. (2012) tested the decolourisation of six azo dyes in various conditions (aerobic and anaerobic). The decolourisation results depended on the dye structure, but in the cases of methyl orange, orange 1 and Congo Red, the final decolourisation was higher under anaerobic conditions, which confirms the observed tendency in our studies.

The dye mixture was significantly removed by both tested strains from the beginning of the experiment (Fig. 2a–c). After 1 h, both strains decolourised the mixture by ∼80 % in all conditions. Finally, the living biomass of the strain Rz7 removed 89.12 % in the static, 87.58 % in the semistatic and 96 % in the shaken conditions. The strain Bz4 removed 99.04, 89.62 and 77.12 %, respectively. High sorption of the dye mixture was observed for both tested strains. The dead biomass of the strain Bz4 adsorbed more than 70 % after 1 h, whereas the dead biomass of the strain Rz7 adsorbed approximately 60 %. Finally, both strains adsorbed approximately 75 % of the dye mixture (Fig. 2c).

The UV–Vis scan of the dyes, which was shown in our previous publication (Zabłocka-Godlewska et al. 2014), and results obtained with the dead biomass, suggests that the dyes in the mixture interacted with one another. The structure of the dyes in the mixture was most likely changed, which confirms the shift in wavelength of the maximal absorbance compared to those of the single dyes. This interaction may affect the mixture properties. High adsorption of single dyes by the strain Bz4 was observed, whereas the strain Rz7 did not adsorb the tested dyes at such a high level. When the dyes were used in the mixture, the adsorption by both tested strains was significant. The mixture was finally removed by 75.28 and 75.86 % by the dead biomass of Bz4 and Rz7, respectively. Such high adsorption by the dead biomass of the strain Rz7 was observed from the beginning of the experiment (61.03 % after 1 h), whereas the single-dye adsorption was below 30 %. This result confirms the changes of dye structure in the mixture. Differences in decolourisation of the dye mixture and single dyes were also observed for fungal strains, which were studied by Przystaś et al. (2013).

It is necessary to mention that the dyes in the prepared mixture belong to different classes. Most studies concentrated on one group of dyes. Saratale et al. (2010) studied the decolourisation of azo dye mixtures under static conditions by a consortium of *Proteus vulgaris* and *Micrococcus glutamicus*. After 72 h, a mixture of three dyes was 86 % removed, the second mixture was 92 % removed, and a mixture of six dyes was 74 % removed. Jadhav et al. (2010) showed that the textile effluent was completely decolourised in static conditions after 48 h by a bacterial consortium (two *Pseudomonas* strains). One of the strains in this consortium was previously studied by Kalyani et al. (2009). A mixture of ten azo dyes was removed by the strain *Pseudomonas* sp. SUK1 by approximately 25 % after 4 h and approximately 90 % after 24 h in static conditions. As observed, all studies with dye mixtures were only performed in static conditions, which were recommended for azo dye removal.

### Toxicity Evaluation

High decolourisation effectiveness by the living biomass of Bz4 corresponded with the decrease in toxicity to *Daphnia magna* (from toxicity class IV to class III for EB, from V to IV for BG and from V to III or IV for the dye mixture). In case of the strain Rz7, the decrease in zootoxicity was only noticed in samples with single dyes (decrease of one class of toxicity) and the mixture that was incubated in shaken conditions (Table 2). The biological transformation of dyes by both *Klebsiella* strains leads to the formation of less toxic products than dyes. The participation of transformation processes in decreasing the toxicity confirms the following obtained results for the samples with dead biomass.

**Table 2:** Results of the toxicity tests

Strain	Culture conditions		*Daphnia magna*			*Lemna minor*		
			EC_50_	TUa	Toxicity class	EC_50_	TUa	Toxicity class
Bz4	Static	EB	30.03 ± 2.07	3.33	III	35.7 ± 1.96	2.80	III
		BG	6.09 ± 0.27	16.42	IV	41.49 ± 0.46	2.41	III
		Mix	14.83 ± 0.19	6.74	III	18.76 ± 0.79	5.33	III
	Semistatic	EB	21.41 ± 1.08	4.67	III	23.04 ± 0.69	4.34	III
		BG	2.64 ± 0.17	37.9	IV	19.23 ± 1.08	5.2	III
		Mix	11.98 ± 0.27	8.35	III	21.88 ± 1.44	4.57	III
	Shaken	EB	13.64 ± 0.70	7.33	III	6.25 ± 1.1	16.00	IV
		BG	2.10 ± 0.14	47.58	IV	15.50 ± 1.1	6.45	III
		Mix	9.63 ± 1.14	10.38	IV	23.58 ± 6.20	4.24	III
	Dead biomass	EB	2.31 ± 0.09	43.29	IV	13.64 ± 1.14	7.33	III
		BG	24.15 ± 1.10	4.14	IV	21.37 ± 1.1	4.68	III
		Mix	n.d.	n.d.	V	10.63 ± 1.2	9.41	III
Rz7	Static	EB	39.53 ± 2.50	2.53	III	22.52 ± 0.46	4.44	III
		BG	1.15 ± 0.17	86.957	IV	20.70 ± 0.51	4.83	III
		Mix	n.d.	n.d.	V	5.71 ± 0.27	17.5	IV
	Semistatic	EB	62.50 ± 0.09	1.6	III	16.81 ± 1.14	5.95	III
		BG	1.33 ± 0.02	75.05	IV	20.75 ± 0.95	4.82	III
		Mix	n.d.	n.d.	V	64.10 ± 4.23	1.56	IV
	Shaken	EB	75.19 ± 3.20	1.33	III	25.00 ± 1.7	4.00	III
		BG	3.67 ± 0.20	27.22	IV	16.81 ± 0.7	5.95	III
		Mix	9.35 ± 1.1	10.7	IV	4.40 ± 1.1	22.73	IV
	Dead biomass	EB	37.45 ± 1.80	2.67	III	22.78 ± 1.50	4.39	III
		BG	0.85 ± 0.10	117.64	V	25.45 ± 0.52	3.93	III
		Mix	n.d.	n.d.	V	7.81 ± 1.10	12.80	IV
Controls		EB	9.43 ± 0.22	10.62	IV	22.22 ± 2.10	4.5	III
		BG	0.98 ± 0.07	102	V	3.07 ± 0.52	32.58	IV
		Mix	0.92 ± 0.15	108.7	V	1.20 ± 0.01	83.3	IV

*n.d.* not detected

Despite the high effectiveness of adsorption on the dead biomass of the strain Bz4, the decrease of zootoxicity was only observed in the samples with BG. In the samples with Rz7, the zootoxicity only decreased for EB. In other samples with single dyes and their mixture, the zootoxicity remained constant (Table 2). The obtained results for EB are comparable with those presented in our previous study (Zabłocka-Godlewska et al. 2012). The zootoxicity of Evans blue decreased for the tested *Pseudomonas* strain SDz3 regardless of the process conditions. Only in the case of the strain Sz6, the zootoxicity increased in static conditions. In the samples with dead biomass, the toxicity increased. *Staphylococcus arlettae* decolourisation ability and zootoxicity of the end-products were studied by Franciscon et al. (2009a). They noticed a lack of zootoxicity in aerobic conditions and significant decrease in microaerophilic samples. These authors tested the decolourisation of the same azo dyes by *Klebsiella* (Franciscon et al. 2009b) and observed a decrease in mortality of *Daphnia magna* for all four tested dyes in aerobic conditions and for three of them in microaerophilic conditions. For Direct Blue 71, higher mortality was observed in the microaerophilic samples. The authors suggested that it was related to the higher amount of aromatic amines that were produced during the biodegradation of this triazo dye. Higher zootoxicity was observed in microaerophilic samples, which was related to the accumulation of aromatic amines that were produced in the anaerobic first stage of the bacterial azo dye decolourisation process. The zootoxicity of the decolourisation products of brilliant green was examined in our previous study (Przystaś et al. 2012). In all samples regardless of incubation conditions, the zootoxicity decreased. In the samples with living bacterial biomass, the toxicity even decreased from class V to class III.

The phytotoxicity was tested on *Lemna minor*. In the samples with EB, no change in toxicity was observed, and in the shaken samples with Bz4, the toxicity increased from class III to class IV (Table 2). In other samples, the phytotoxicity decreased. In our previous study (Przystaś et al. 2012), an identical situation (no change in toxicity) was noticed for the strain *Burkholderia cepacia*. For the *Pseudomonas* strains (Zabłocka-Godlewska et al. 2012), the phytotoxicity only decreased in the static and semistatic samples. In other samples, no change was noticed. The brilliant green removal by *Klebsiella* strains decreases the phytotoxicity (from IV to III; Table 2). These results contradict our previous study (Przystaś et al. 2012), where the decrease was only observed in the sample with *Chryseomonas luteola* in static conditions. The phytotoxicity of the dye mixture only decreased for the strain Bz4 regardless of the modifications of decolourisation conditions. High decolourisation effectiveness by Rz7 had no significant effect on the change in phytotoxicity class (Table 2). The phytotoxicity results obtained for the dye mixture suggest a significant effect of the sorption properties of the strain that is used. In the case of the strain Bz4, which has a strong sorption capacity, the phytotoxicity decreased. In case of the strain Rz7, which is characterised by weaker sorption properties, the phytotoxicity remained unchanged, but the TUa value decreased. The obtained results with dead biomass confirm that the adsorption on biomass may strongly reduce the phytotoxicity of the dye mixture. The decrease in phytotoxicity was only observed in samples with the strain Bz4, which has a high adsorption capacity. The phytotoxicity of TPM methyl red decolourisation products was tested by Cui et al. (2012). Regardless of the process conditions (aerobic and anaerobic), the phytotoxicity decreased. In comparison with the control (no germination), after decolourisation, 90 and 70 % of *Brassica pekinensis* seed germination was observed in aerobic and anaerobic samples, respectively. Wu et al. (2012) used *Shewanella oneidensis* to decolourise TPM aniline blue. The decrease in phytotoxicity was noticed particularly for the length of the shoot of *Oryza sativa*. A significant decrease in genotoxicity after decolourisation was also proven.

## Conclusions

Both tested *Klebsiella* strains have a significant decolourisation potential for the dyes of different classes (fluorone, azo and triphenylmethane class). The tested bacteria have a great potential to remove BG and EB as single substances and mixtures. The dye concentration and type affect the decolourisation effectiveness. The best dye removal results were obtained for Bengal rose, and the worst result was obtained for crystal violet. The strain Bz4 was more sensitive to higher concentrations of crystal violet and brilliant green. The brilliant green decolourisation in the concentration test had higher efficacy than the removal of Evans blue and dye mixture. Between two tested strains, differences in dye removal potential were observed. The best results were obtained for the strain Bz4 in the concentration test and main experiment for the samples with Evans blue and the dye mixture regardless of the process conditions. In the main experiment with brilliant green, the best results were obtained for the strain Rz7, which is similar to the results of the decolourisation potential test. The concentration test shows worse removal results of brilliant green and Evans blue than the potential tests (at identical dye concentrations), which was related to different volumes of medium in the tests, whereas the inoculum volume was identical.

High adsorption of dyes by the biomass of the strain Bz4 was observed from the beginning of the experiment, whereas for the strain Rz7, this situation was only observed in the samples with the dye mixture. The process conditions significantly affect the rate of decolourisation effectiveness for both tested strains. The best removal of azo Evans blue and the dye mixture was obtained in static and semistatic samples, and the best removal of TPM brilliant green was obtained in the static samples. The zootoxicity decreased in all samples with living biomass of the strain Bz4 but only in the samples with single dyes for the strain Rz7. The phytotoxicity only decreased for the strain Bz4 in the samples with BG and dye mixture and for strain Rz7 only in the samples with BG. The process conditions did not affect the changes in sample toxicity. The high decolourisation potential of both *Klebsiella* strains is related to the reduction in toxicity class, which suggests the possibility of their application in larger scale.
